# Sequencing of the Cheese Microbiome and Its Relevance to Industry

**DOI:** 10.3389/fmicb.2018.01020

**Published:** 2018-05-23

**Authors:** Bhagya. R. Yeluri Jonnala, Paul L. H. McSweeney, Jeremiah J. Sheehan, Paul D. Cotter

**Affiliations:** ^1^Food and Nutrition Deptartment, University College Cork, Cork, Ireland; ^2^Teagasc Food Research Centre, Fermoy, Ireland; ^3^APC Microbiome Ireland, Cork, Ireland

**Keywords:** high throughput sequencing, cheese, microbiota, sensory characteristics, metatranscriptomics, industry

## Abstract

The microbiota of cheese plays a key role in determining its organoleptic and other physico-chemical properties. It is essential to understand the various contributions, positive or negative, of these microbial components in order to promote the growth of desirable taxa and, thus, characteristics. The recent application of high throughput DNA sequencing (HTS) facilitates an even more accurate identification of these microbes, and their functional properties, and has the potential to reveal those microbes, and associated pathways, responsible for favorable or unfavorable characteristics. This technology also facilitates a detailed analysis of the composition and functional potential of the microbiota of milk, curd, whey, mixed starters, processing environments, and how these contribute to the final cheese microbiota, and associated characteristics. Ultimately, this information can be harnessed by producers to optimize the quality, safety, and commercial value of their products. In this review we highlight a number of key studies in which HTS was employed to study the cheese microbiota, and pay particular attention to those of greatest relevance to industry.

## Introduction

Cheese has a diverse microbial community, which indeed can vary within the cheese from the core to the surface that is greatly influenced by manufacturing including ripening conditions. Understanding the composition of this community (microbiota), and its impact on the quality and safety of cheese products, is of critical importance. In addition to, in the majority of cases, consciously added starter and adjunct bacteria (which are added as a supplement), cheese contains a heterogeneous variety of other, non-starter, microorganisms. These various microorganisms can play vital roles in the development of the organoleptic properties of cheese (Fox et al., 2000), nutrient composition, shelf-life, and safety.

**Figure 1:**
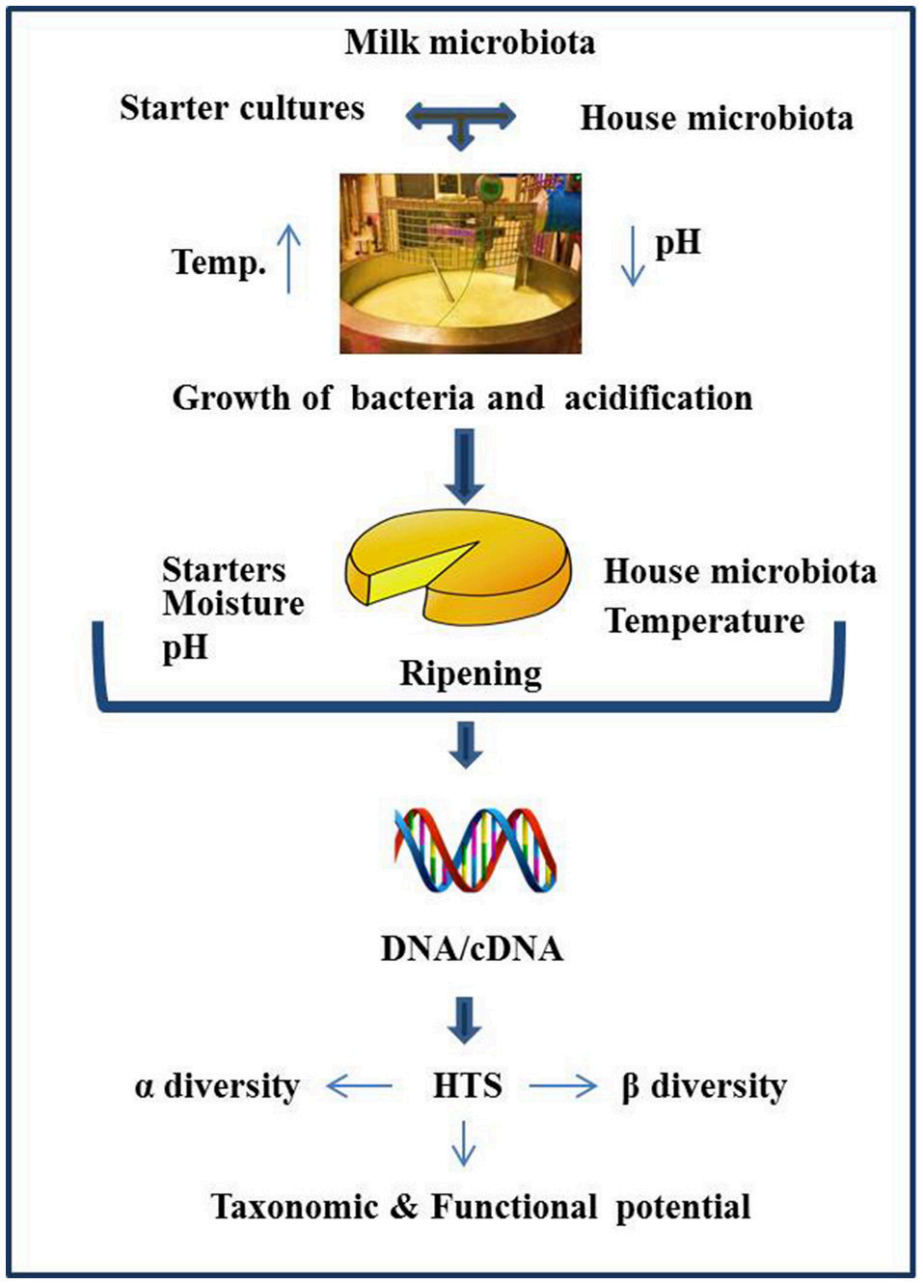
Schematic representation of factors influencing the cheese microbiota, as revealed by HTS.

Historically, culture-based microbiology techniques were used to gain an understanding of the microbial component of cheese. However, it has become increasingly clear that this approach can be limited in its ability to detect “difficult-to-culture” or sub-dominant microorganisms, thereby potentially providing misleading results. As a result, culture-independent approaches have become increasingly popular. These approaches involve DNA, and occasionally RNA, based molecular methods, with high throughput sequencing (HTS) gaining particular attention by virtue of its potential to provide an insight into the total microbial community of the cheese. The application of HTS can provide valuable information relating to the influence of geography, manufacturing processes, climatic conditions, seasonal variation, use of raw or pasteurized milk, and a variety of other factors on the cheese microbiota (Figure 1). The application of HTS for microbiota analysis can involve any of three primary approaches i.e., (1) Amplicon sequencing—whereby a fragment of highly conserved gene, ideally with variable regions therein, is used for sequencing, with comparison to databases allowing taxonomic assignment. The 16S rRNA gene is most frequently used and provides an insight into the bacterial composition of samples (generally to the genus level), (2) Shotgun metagenomic sequencing—which involves non-targeted sequencing of the DNA in a sample and, again on the basis of comparison with databases, can be used to classify all of the microorganisms, i.e., not just bacteria present (to species, or even strain, level) as well as information regarding the functional potential of the community, (3) Metatranscriptomics (RNASeq)—whereby total mRNA in the sample is sequenced (after first being converted to cDNA) to reveal the extent to which different genes are expressed and, in turn, the relative activity of different components of the community. These three experimental procedures differ with respect to library preparation, sequencing strategy, and the approaches taken for bioinformatic analysis. Some of the most common outputs from these analyses relate to α-diversity, β-diversity, and a determination of the relative abundance of different taxa. α-diversity is a measurement of diversity, such as total number of species, within a sample and measures such as the Simpson and Shannon diversity indices are employed. β-diversity reflects differences in diversity across different samples, with Bray-Curtis and UniFrac being among the distance metrics used. For metagenomics and metatranscriptomics, several tools are available for key steps, i.e., binning, assembly (where relevant), and mapping/assignment of sequences obtained (Di Bella et al., 2013; Walsh et al., 2017). There have been a number of research and review papers dedicated to describing methodologies used for sequencing (Di Bella et al., 2013) work flow, limitations, applications to dairy foods (Ercolini, 2013; Walsh et al., 2017), comparing sequencing platforms, and bioinformatics pipelines as well as highlighting pros and cons associated with all of the above (Kelleher et al., 2015; Clooney et al., 2016; Walsh et al., 2018) Depending on sequencing depth, HTS can facilitate the detection of taxa present at low levels, a feature of great value in the context of testing for spoilage or pathogenic microbes. HTS technology can also be employed to study the microbial diversity of protected designation of origin (PDO) cheeses to establish which components are responsible for the authentic taste, flavors, and textures of these products (Alegría et al., 2012; Fuka et al., 2013; De Filippis et al., 2014; Delcenserie et al., 2014; De Pasquale et al., 2014, 2016; Riquelme et al., 2015; Dalmasso et al., 2016; Parente et al., 2016; Giello et al., 2017; Gonçalves Dos Santos et al., 2017; Li et al., 2017). The same technology could, in the future, be employed to establish authenticity. However, as these topics, and studies related to microbial dynamics of food ecosystems, processing environments, and production facilities have been reviewed in depth recently (Mayo et al., 2014; Galimberti et al., 2015; Bokulich et al., 2016; De Filippis et al., 2017; Doyle et al., 2017b; Macori and Cotter, 2018), we are not addressing those topics here. In this review, we specifically focus on providing an overview of studies in which HTS has been applied to provide insights into the microbiota of different cheeses that are of value from an industrial perspective as well as the factors that modulate these microbes (Table 1) and the identity of adventitious and other microbes (Table 2).

**Table 1:** HTS detection of increase and decrease of microbiota by the action of some influencing factors.

**Influencing factor**	**Cheese type**	**Increases/decreases**	**References**
Herbs	Irish artisanal	↑Lactobacilli	Quigley et al., 2012
		↓Lactococci	
Salt	Irish artisanal	↓*Leuconostoc*	Quigley et al., 2012
		↓*Pseudomonas*	
Hay (feed to cows)	Caciocavallo	↑Salty, Sour, Umami	Giello et al., 2017
Silage (feed to cows)	Caciocavallo	↑Tenderness and oiliness	Giello et al., 2017
Wooden surfaces	Cotija	↑*L. mesenteroides*	Escobar-Zepeda et al., 2016
		↑*W. paramesenteroides*	
Temperature & pH	Mozzarella	↑*L. lactis*	De Filippis et al., 2014
	Grana Padano	↑*L. fermentum*	
	Parmigiano Reggiano	↑*S. thermophilus*	
		↑*L. delbrueckii*	
		↑*L. helveticus*	
Moisture	Brie	↑*Galactomyces*	
	Camembert		
	Cloth bound cheddar,	↓*Scopulariopsis*	
	St. Nectaire,	↓*Aspergillus*	Wolfe et al., 2014
	Tomme de Savoie	↓*Actinobacteria*	
		↓*Staphylococcus*	
Previous evening milk (Stored at 10^*o*^C)	Plaisentif	*Lactococcus, Lactobacillus* and *Streptococcus*	Dalmasso et al., 2016
Smoking	Herve	↓Enterobacteria	Alegría et al., 2012
Scalding temperature	Dutch-type Cheese	↑*Lactobacillus* spp.	Porcellato and Skeie, 2016
		↑*Leuconostoc* spp	
Washing of rind	Irish Cheese	↑Lactococci	Quigley et al., 2012
NMC	Italian PDO Cheese	↑*S. thermophilus*	Parente et al., 2016
		↑*L. delbrueckii*	
NWC	Italian PDO Cheese	↑*L. lactis, Lactobacillus fermentum, S. thermophilus, L. delbrueckii* and *Lactobacillus helveticus*	De Filippis et al., 2014
Production practices	Serpa	Yeast population	Gonçalves Dos Santos et al., 2017
↑Temperature	Caciocavallo Silano	↑NSLAB	De Filippis et al., 2016
↓Humidity			

Abundance of bacteria in different cheese samples was detected by HST, ↑ (increase) and ↓ (decrease) indicates levels of certain bacteria as influenced by factors stated

**Table 2:** Adventitious, previously overlooked, and spoilage bacteria identified by HTS analysis of different cheese matrix and dairy environment.

**Bacteria**	**Cheese type**	**Function**	**References**
**ADVENTITIOUS**			
*Pseudoalteromonas* spp.	Cheese stored at low temperature	Flavor compounds	Wolfe et al., 2014
*Lactic acid bacterial strains*	Cotija	Bacteriocins for biopreservation	Escobar-Zepeda et al., 2016
*Geotrichum candidum*	Camemberti	Aroma, texture	Lessard et al., 2014
*Penicillium camemberti*			
*Brevibacterium linens*	Raw milk cheese	Protein and fat metabolism	Frétin et al., 2018
*Staphylococcus equorum*			
*Lactococcus lactis*			
*Lactococcus chungangensis/raffinolactis*			
*Lactobacillus casei/paracasei*			
*Lactococcus*	Dutch-type Cheese	Flavor	Porcellato and Skeie, 2016
*S. thermophilus*	Mozzarella, Grana Padano, Parmigiano Reggiano	*lacS* gene	De Filippis et al., 2014
*S. thermophilus*	Undefined milk starter	*serB* gene	Parente et al., 2016
Mesophilic lactobacilli	Fiore Sardo	Secondary proteolysis, esters, alcohols, aldehydes, sulfur compounds	De Pasquale et al., 2016
	Pecorino Siciliano		
	Pecorino Toscano		
Thermophilic LAB	Percorino Siciliano	Free amino acids	De Pasquale et al., 2016
*Brevibacterium*	Percorino Toscano	dimethyl trisulphide, dimethyl disulphide, methional, S-methyl thioesters	De Pasquale et al., 2016
*B. linens*	Surface ripened Cheese	Sulphur compounds, 2-methyl-1-butanol	Bertuzzi et al., 2018
*Geotrichum candidum*			
*Staphylococcus xylosus*			
*Debaryomyces hansenii*	Surface ripened Cheese	Alcohols	Bertuzzi et al., 2018
*Glutamicibacter arilaitensis*	Surface ripened Cheese	Ketones, alcohols, acids	Bertuzzi et al., 2018
*Acetobacter*	Kazak	VOC, amino acids, fatty acids	Zheng et al., 2018
*Lactococcus*			
*Bacillus*			
*Staphylococcus*			
*Kurthia, Moraxella*.			
*Dipodascus*,			
*Pichia, Penicillium*,			
*Issatchenkia, Candida*			
*Lactococcus*	Canestrato Pugliese	Degrade carbon sources	De Pasquale et al., 2014
*Pseudoalteromonas*	Washed rind Cheese	MGL	Wolfe et al., 2014
LAB	Cotija	Bacteriocins	Escobar-Zepeda et al., 2016
*G. candidum*	Camembert	Sensory characteristics	Lessard et al., 2014
*Penicillium camemberti*			
*S. thermophilus*	Reblochon-style French Cheese	Protein folding, Sorting, degradation, signal transduction	Monnet et al., 2016
*L. delbrueckii*	Reblochon-style French Cheese	Transcription, Translation, nucleotide and lipid metabolism	Monnet et al., 2016
*G. candidum*	Reblochon-style French Cheese	Lipid and amino acid metabolism	Monnet et al., 2016
*D. hansenii*	Reblochon-style French Cheese	Other amino acids metabolism	Monnet et al., 2016
*L. lactis*	Surface ripened Cheese	Lactose metabolism, Lipolysis, Proteolysis, catabolism of amino acids and fatty acids	Dugat-Bony et al., 2015
*Kluyveromyces lactis*			
*D. hansenii*			
*G. candidum*			
*Corynebacterium casei*			
*Hafnia alvei*			
**PREVIOUSLY OVERLOOKED**			
*Exiguobacterium*	Queso Fresco		Lusk et al., 2012
*Flavobacterium*	Plaisentif		Dalmasso et al., 2016
*Brevibacterium*			
*Salinicoccus*			
*Vagococcus*			
*Anaerobacillus*			
*Sphingobacterium*			
*Klebsiella*			
*Dehalobacter*	Cotija		Escobar-Zepeda et al., 2016
*Desulfohalobium*			
*Halomonas*			
*Thermohalobacter*			
*Haloquadratum*			
*Prevotella*	Irish artisanal cheese		Quigley et al., 2012
*Faecalibacterium*			
*Idiomarina*	Brine		Marino et al., 2017
GNO2			
TM7			
*Tremellomycetes*	Wooden Shelves		Guzzon et al., 2017
**SPOILAGE/CONTAMINANTS**			
*C. tyrobutyricum*	Grana Padano	Blowing defect	Bassi et al., 2015
*C. butyricum*			
*Klebsiella*	Plaisentif	Poor hygiene, Contaminants of Boilers	Dalmasso et al., 2016
*Morganella*			
*Erwinia*			
*Acinetobacter*			
*Brevibacterium*	Smear ripened Cheese	Red-brown defect	Guzzon et al., 2017
*Corynebacterium*			
*Microbacterium*			
*Enterococcus faecalis*	Herve	Contaminant	Delcenserie et al., 2014
*Thermus thermophilus*	Pink defective cheese	Pink color	Quigley et al., 2016
*Escherichia* spp.	NWC	Contaminants	De Filippis et al., 2014
*Enterobacter cowanii*			
*Enterobacteriaceae*			
*Agrobacterium* spp.			
*Alicyclobacillus* spp.			
*Propionibacterium acnes*			
*Lactobacillus curvatus*	Irish artisanal cheeses	Production of tyramine	
*Enterococcus faecium*	Reblochon, Morbier,		
*Enterococcus faecalis*	Tête deMoine,	Production of Histamine	O'Sullivan et al., 2015b
*Lactobacillus buchneri*	Pecorino Sardo		

## Identification of factors influencing the development of the cheese microbiota

Microbial composition and diversity differs from raw to pasteurized milk, and between curd, whey, and cheese. The raw milk microbiota is influenced by microbes present in the teat canal, the surface of teat skin, hygiene practices, animal handlers, and the indigenous microbiota of equipment and storage containers. The origin of the milk would also seem to influence the levels of diversity therein, with cow's milk appearing to be more diverse than that from goats and sheep (Quigley et al., 2012). The type of grazing system employed, i.e., extensive or semi-extensive, affects the dynamics of teat skin microbiota and thus, in turn, the microbes in raw milk cheeses. Indeed, in one study, 27% of bacteria detected in raw milk cheese were also found on the teat surface. These bacteria included species involved in the production of flavor, aroma, and color development, such as *Brevibacterium linens, Staphylococcus equorum*, and Lactic Acid Bacteria (LAB) such as *Lactococcus lactis, Lactococcus chungangensis/raffinolactis*, and *Lactobacillus casei/paracasei*, which can contribute to protein and fat metabolism (Frétin et al., 2018). The sensory attributes of cheese can also be influenced by the type of feed (e.g., hay, silage) given to cows. Indeed, this was recently demonstrated in a study of Caciocavallo cheese where salty, sour, bitter, and umami flavors were lower in “hay”-cheese compared to “silage”-cheese, and higher levels of tenderness and oiliness were also evident in “silage”-cheese relative to “hay”-cheese (Giello et al., 2017). High temperature treatment (when pasteurized milk is used) and low pH also contribute to the selection of specific bacteria in some artisanal cheeses that are made in the absence of starter bacteria (De Filippis et al., 2014). These and other factors, such as salt content, degrees of ripening, addition of ingredients like herbs, and spices also influence the cheese microbiota and, in turn, organoleptic properties of cheese. The influence of such factors was demonstrated in one representative study of Irish artisanal cheeses where, for example, proportions of lactobacilli increased and lactococci decreased due to the addition of herbs, while high salt content suppressed the growth of *Leuconostoc* and *Pseudomonas* (Quigley et al., 2012).

The microbial profile of cheeses can differ between producers, according to the process of production and the inclusion, or absence, of starters. Plaisentif is a traditional Italian cheese made from fresh full-fat raw cow's milk without an added starter but, rather, milk from the previous evening (kept at <10°C) and bovine liquid rennet are employed. HTS analyses conducted on samples from nine different producers revealed the presence of the genera *Lactococcus, Lactobacillus*, and *Streptococcus* in the core of all samples but also established that these are present in different proportions and are responsible for variations in the niche-specific characteristics of Plaisentif cheese (Dalmasso et al., 2016). Greater homogeneity was apparent in a recent study involving Herve, a PDO cheese from Belgium. In the past, Herve was made from raw milk but, for safety reasons due to the possible presence of *Listeria monocytogenes*, pasteurized milk has begun to be used. The 16S rDNA analysis showed that 95% of the microbial composition was same in both raw and pasteurized cheese; hence the characters of both forms of the cheese are similar. This might be because of consistency in the manufacturing process across artisanal producers (Delcenserie et al., 2014). The impact of temperature was also evident when the scalding temperature during the manufacture of Dutch-type cheeses was increased from 37 to 39°C. This change resulted in an increase in *Lactobacillus* sp. and *Leuconostoc* sp. during ripening. This change also promoted flavor formation by lysing lactococci, which release enzymes like peptidases (Porcellato and Skeie, 2016). The influence of manufacturing processes on bacterial communities was also noted in the case of Oscypek cheese. In that instance, a decrease in the counts of enterobacteria was apparent from the curd to the smoked cheese as a result of the smoking process, thus improving the quality and safety of the traditional product (Alegría et al., 2012). Cotija is another example of a cheese in which fluctuations in composition have been observed. This handmade Mexican cheese is prepared from raw milk with no added starters. Ripening of Cotija is performed in an open environment and, thus, humidity, rain, and temperature are important parameters to be considered. Furthermore, the wooden surfaces used for kneading the curd while salting, vats, and other tables used in the process of cheese-making all act as a source of the microbes found in this cheese. About 80% of the bacterial population of Cotija was consisted of a combination of *Lactobacillus plantarum, Leuconostoc mesenteroides*, and *Weissella paramesenteroides*. These species are thought to be responsible for the development of authentic flavor compounds arising from their lipolytic and proteolytic activity during ripening (Escobar-Zepeda et al., 2016). It is thought that the wooden surfaces are most likely the source of the *Leuconostoc* and *Weissella* species present (Settanni et al., 2012). It is important to note, however, that even when cheeses are produced on a larger industrial scale, including the use of starters to minimize variability, differences in the associated microbiota can be seen. This was apparent from a study of different batches of brine-salted continental-type cheeses made during the same day of production. In this study, 16S rRNA sequencing revealed that cheese produced later in the production day had a more diverse bacterial composition than cheese produced earlier, possibly due to the accumulation of bacteria in the system before cleaning-in-place (CIP) processes are employed at the end of the manufacturing day (O'Sullivan et al., 2015a).

Cheese rinds have a very complex microbiota when compared to core samples; this microbiota varies between types of rinds, degrees of ripening, and the environmental conditions. HTS analyzes of 11 artisanal Irish cheeses (soft, hard, and semi hard) revealed the presence of 19 genera, of which *Lactococcus, Leuconostoc*, and *Lactobacillus* were in both rind and core samples. *Corynebacterium, Facklamia, Flavobacterium*, and *Cronobacter* were detected in rind samples only. The relative proportions of lactococci were found to be high in naturally ripened rinds relative to smear/washed rinds. This is an ultimate result of the washing of rind in that cheese (Quigley et al., 2012). In one seminal study, 137 different rind samples that included bloomy, natural, and washed types from 10 countries were examined using HTS to understand the mechanisms of how microbial communities form multispecies biofilms. It was noted that rind samples from cheeses made at different geographical regions showed similar patterns of microbial clusters, showing that, in this instance, distance did not have impact on microbial community. Some of the specific patterns noted were as follows; the fungus *Galactomyces*, which is responsible for the formation of a dense white rind on bloomy rind cheeses (e.g., Brie, Camembert), is positively correlated with moisture. Natural rind cheeses (e.g., Clothbound Cheddar, St. Nectaire, and Tomme de Savoie) are abundant in fungi and bacteria, such as *Scopulariopsis, Aspergillus, Actinobacteria*, and *Staphylococcus*, which are negatively correlated with moisture. The microbial composition of washed rind cheese (e.g., Gruyere, Epoisses) was found to be a mix between that of bloomy and natural rinds (Wolfe et al., 2014). Finally with regard to cheese rinds, it is also worth noting that HTS is beginning to be combined with other techniques, such as microscopy, to identify the fungal and bacterial interactions, and the bacterial dispersal on fungal networks (Zhang et al., 2018).

As already noted above, many artisanal cheeses are produced without the addition of starter cultures. Undefined natural milk starter cultures (NMC), produced by heat treatment of raw milk (60–63°C, 20–30 min) followed by incubation at high temperatures (39–42°C), are used to make some Italian PDO cheeses. Such cultures, prepared by backslopping (i.e., inoculating milk with a previous batch of the culture) using raw milk collected from pasta filata cheese plants, were found to be dominated by *Streptococcus thermophilus* and *Lactobacillus delbrueckii* (Parente et al., 2016). Natural whey cultures (NWC), produced by incubating the whey from previous batches of cheese production at high temperature (39–54°C), is also used to make some PDO Italian cheeses. These NWCs were dominated by *L. lactis, Lactobacillus fermentum, S. thermophilus, L. delbrueckii*, and *Lactobacillus helveticus*, though the abundance of these species was found to be significantly different across the three cheese types, Mozzarella, Grana Padano, Parmigiano Reggiano (De Filippis et al., 2014). The combined pressure of temperature and pH selects for these bacteria. In the case of high-moisture Mozzarella cheese, the mode of acidification, i.e., through addition of citric acid, by starters, or by using undefined starters, greatly influences the growth of the dominant microbial components, thereby differentiating cheeses produced by different dairies (Guidone et al., 2016). This study demonstrated that HTS can be used by Mozzarella producers to identify the dominant bacteria present and, in turn, helps them to choose the mode of acidification that is best suited to obtaining specific desirable characteristics. Pico is an artisanal Azorean cheese, made from raw milk, animal rennet, and salt without any starters. HTS analysis of this cheese by Riquelme et al. (2015) established that Pico manufacture is a *Lactococcus*-driven process with contributions from accompanying *Lactobacillus* and *Gammaproteobacteria* populations. The availability of this information is useful in terms of selecting starter/adjunct cultures specific for Pico cheese, as well as highlighting some hygienic and safety measures that need to be taken by industries to improve the shelf life of these cheeses (Riquelme et al., 2015). From a safety perspective, HTS detection of microbes in Danish raw milk found that *L. helveticus, S. thermophiles*, and *L. lactis* are dominant (Masoud et al., 2011) and, following spiking with *Listeria innocua* and *Staphylococcus aureus* during manufacture of this cheese to detect the fate of pathogenic bacteria during ripening, it was found that these species were absent from the ripened cheese. The control of these microbes was presumed to be due to acidification, environmental conditions or antimicrobials produced by the LAB (Masoud et al., 2012).

Serpa is an artisanal PDO Portuguese cheese, made from raw ewe's milk using aqueous infusion of *Cynara cardunculus* L. (artichoke), without starters. The fungal communities in this cheese are thought to have a key role in developing the associated organoleptic properties. HTS analysis of this fungal community of both PDO and non-PDO forms of Serpa cheeses, highlighted diversity among the yeasts present in a manner suggested that production practices and the associated environment have a considerable influence on the final yeast population. Through these investigations it was possible to alter ripening conditions to favor the dominance of yeast species that are desirable for optimal Serpa production (Gonçalves Dos Santos et al., 2017). Another ewe's milk cheese produced by traditional techniques without starters, in this case from Croatia, has also been the subject of HTS-based analysis. This revealed the presence of pathogenic bacteria in fresh milk and cheese. However, their abundance was low at the end of ripening, highlighting the importance of ripening for 90 days to avoid problems related to the safety of the cheese (Fuka et al., 2013). This approach highlighted the merits of using HTS to amend production processes to alter quality and safety, particularly in instances where neither a starter nor pasteurized milk are used. An alternative, HTS-based approach to studying the microbiota of cheese has been to use single molecular real-time (SMRT) sequencing, a third generation technique that provides longer DNA reads. This technique has been used to sequence a Kazakhstan cheese made using NWC as starters, revealing that *L. lactis, L. helveticus, S. thermophiles*, and *Lactobacillus bulgaricus* were the dominant species in this cheese. Differences between the microbiota of this cheese and artisanal cheeses from Belgium, Italy, and Kalmykia (Li et al., 2017) likely explain the varying characteristics of these cheeses.

The house microbiota that colonize the equipment, brine tanks, vats, wooden surfaces, knives, and other surfaces in production facilities can play a key role in shaping the microbial communities of cheese. This colonization depends on the characteristics of the surfaces, nutrient availability and composition, ability of microbes to form biofilms, ecological factors, and on operators and cleaning processes (Stellato et al., 2015). Studies focusing on environmental microbiota have been conducted on cheese plants in which one company produces two different cheese types (Calasso et al., 2016) and, in another instance, two different facilities that produce the same type of cheese (Bokulich and Mills, 2013). In the former case, it was deduced that *S. thermophilus* colonized, to different degrees, almost all surfaces in the dairy plant and acted as an indirect source of starter inoculation (Calasso et al., 2016). In the latter study, it was revealed that the type of processing facility and selective forces in the environment, such as the temperature in the cheese plants, influenced the growth of specific house microbiota, which were in turn responsible for the chemosensory properties of the artisanal cheese (Bokulich and Mills, 2013).

One of the factors that can contribute to differences across different production facilities is brining. The microbial diversity and composition of brine depends on the type of cheese made, the specific cheese plant and on salinity concentrations. Adventitious bacteria present in brine, such as *Staphylococcus equorum*, which can have a strong antibacterial activity against *L. monocytogenes* on cheese surfaces, can play important roles in the development of the flavor and color properties of smear ripened cheeses. However, contaminated brine can in turn contaminate the cheese core and surface, with Mozzarella cheese being among the cheeses that are susceptible to such spoilage. Indeed, as a consequence, it has been suggested that sanitization of brine should be prioritized and that brine should be checked regularly for the presence of contaminants (Marino et al., 2017). Cleaning is an important factor to eliminate spoilage bacteria present on the surfaces in dairy plant, with the type of surface material having a key influence on bacterial adherence. Surface materials such as plastic, in the case of gaskets used for the molding of some cheese, is more porous and not suitable for thorough cleaning as it is sensitive to hot water, which causes corrosion and increases the possibility of bacterial adherence (Stellato et al., 2015). As a consequence, it is recommended that steel be used where possible to enhance the safety of products. The importance of proper washing during the manufacture of fermented foods was also highlighted by Lee et al. (2017), who reported that pathogens were replaced by harmless bacteria, such as LAB, as a result of washing. It has been speculated that an equilibrium may exist between the dairy food production and processing environments and the resultant products, with microbial transfer occurring in both directions, which can affect processing dynamics and the quality of the final products (Stellato et al., 2015).

## Identification of previously overlooked bacteria in cheese

Depending on the depth of sequencing, HTS-based taxonomic analysis of cheese can detect microbes present at very low levels (O'Sullivan et al., 2015b; Cotter and Beresford, 2017), which would previously have been overlooked. While, in many cases, it is not yet clear what roles these microbes play, if any, in the context of cheese microbiology, an initial awareness of their presence can lead to further investigations. Here we are highlighting some such subdominant populations as revealed in a selection of representative studies. In one such study, a subdominant taxon was identified after culture-based enrichment. Enriching a food sample in broth for 24 h followed by selective agar plating is a traditional method to isolate subdominant bacteria, and is an especially relevant technique for the detection of foodborne pathogens, in the sample. However, overnight growth in non-selective broth can result in a bias toward the detection of fast-growing, relative to slow-growing, bacteria. In this instance HTS of overnight enriched Queso Fresco cheese sample did successfully reveal the presence of *Exiguobacterium*, which at that point had not previously been documented in cheese (Lusk et al., 2012). It should be noted, however, that the majority of HTS studies take place without an enrichment step. In one typical study, it was noted that Plaisentif cheese contains some rare genera, representing 0.01–0.0001% of total reads, that correspond with *Flavobacterium, Brevibacterium, Salinicoccus, Vagococcus, Anaerobacillus, Sphingobacterium*, and *Klebsiella*, among others (Dalmasso et al., 2016), with *Klebsiella* in particular being regarded as indicative of poor hygiene conditions during manufacturing. In contrast, sub-dominant genera found in Cotija cheese include the halophilic microbes *Dehalobacter, Desulfohalobium, Halomonas, Thermohalobacter*, and *Haloquadratum* (Escobar-Zepeda et al., 2016). Anaerobic bacteria, such as *Prevotella* and *Faecalibacterium*, that are more typically associated with gastrointestinal environments, were for the first time identified in Irish artisanal cheeses at low levels in 2012 (Quigley et al., 2012). Some other recent “firsts” include the detection of *Idiomarina* and two candidate divisions, namely GNO2 and TM7, in brine samples. Of these, *Idiomarina* is consider to be a contaminant of brine, with salt acting as the source for this bacteria (Marino et al., 2017). *Tremellomycetes*, also known as jelly fungi, were also recently found on wooden shelves used for ripening (Guzzon et al., 2017). While the importance of many of these microbes in the context of cheese microbiology is not clear, the fact that HTS has detected the presence of these and other previously-overlooked taxa means that further investigations of such taxa in cheese can now take place.

## Biotype diversity and functional potential of cheese

Assessments of biotype diversity among a species enable researchers to detect variation at sub-species level. This can be achieved by HTS analysis of species-specific genes. One approach to determine biotype diversity involved analyzing a species specific gene with sequence heterogeneity across three Italian cheeses, i.e., Mozzarella, Grana Padano, and Parmigiano Reggiano. *S. thermophilus* was the most abundant bacterium in the three cheeses and, to assess species level diversity, the *lacS* gene, encoding a lactose permease, was selected for specific analysis. Ultimately, 28 different sequence types were identified among which 13 were present at relative abundance > 1%. Mutations at 60 positions identified in the promoter region upstream of *lacS* allowed for further differentiation among the 28 sequence types (De Filippis et al., 2014). A similar sub-species differentiation of *S. thermophilus* has been performed, but instead using the phosphoserine phosphatase gene (serB) as a target sequence. In this case, the approach highlighted the presence of six different sequence types in an undefined milk starter culture (Parente et al., 2016).

Shotgun metagenomic sequencing followed by bioinformatic analysis provides the opportunity to study specific pathways encoded within the cheese microbiome. Ultimately, this technology provides an insight into the functional potential of cheese microbes by studying genes that encode enzymes involved in the catabolic and conversion reactions of amino acids relating to the development of flavor, aroma, and a broad range of other features of relevance to cheese physics, chemistry, and biology. With regard to microbial function or functional potential, spatial distribution analysis of the metabolically active microbiota of three Italian PDO cheeses, Fiore Sardo, Pecorino Siciliano, and Pecorino Toscano, showed a correlation between mesophilic lactobacilli (*L. plantarum*) and secondary proteolysis as well as the synthesis of volatile components (VOC), such as esters, alcohols, aldehydes, and sulfur compounds. Thermophilic LAB found in Pecorino Siciliano cheese correlated with total free amino acid (FAA) concentrations and *Brevibacterium* present on the surface of Pecorino Toscano were found to be related to the synthesis of volatile sulfur compounds, such dimethyl trisulphide, dimethyl disulphide, methional, and S-methyl thioesters (De Pasquale et al., 2016). Shotgun sequencing also revealed a strong correlation between bacteria present in commercially available smear cultures and VOC in surface ripened cheeses. In these instances *B. linens, Geotrichum candidum*, and *Staphylococcus xylosus* correlated with levels of sulfur compounds and 2-methyl-1-butanol, *Debaryomyces hansenii* correlated with levels of alcohols and *Glutamicibacter arilaitensis* with ketones, alcohols, and acids (Bertuzzi et al., 2018). Similarly, a study on Kazak, an artisanal cheese made from fermentation of fresh cow milk through a process involving goat skin bags, identified positive and negative correlations between the core microbiota and amino acid, VOC, and fatty acid concentrations. Kazak cheese analysis revealed that the amino acids, glutamic acid (Glu), histidine (His), isoleucine (Ile), and proline (Pro) had higher positive correlation with one or more abundant bacteria, i.e., *Acetobacter, Lactococcus, Bacillus, Staphylococcus, Kurthia*, and *Moraxella*. Among fungi, *Dipodascus* correlated with concentrations of 9-octadecenoic acid. *Pichia* and *Penicillium* correlated with (Z)-9-hexadecenoic acid, *Issatchenkia* and *Candida* were correlated with leucine (Leu) and phenylalanine (Phe) levels. Various correlations with VOCs were also evident (Zheng et al., 2018). Through harnessing of this knowledge it may be possible to enhance flavor and fat content by choosing specific strains for cheese manufacture. Another multi-omic study focussed on Canestrato Pugliese, an Italian PDO cheese made from raw ewe's milk. HTS revealed that *Lactococcus* dominated within 3 days of ripening and showed a high capacity to degrade carbon sources. Over the ripening period mesophilic lactobacilli, responsible for proteolysis, increased. As part of the study, the authors developed a model system using HTS together with the Biolog Eco-microplate phenotype method that was designed to select adventitious non-starter lactic acid bacteria (NSLAB) to use as adjunct cultures that guarantee high quality standards for traditional cheese (De Pasquale et al., 2014). A shotgun metagenomic sequencing approach was also employed in the pioneering study of washed rind cheese samples from Europe and USA referred to previously. The metabolism of cysteine and methionine produce volatile sulfur compounds such as methanethiol, and the degradation of valine, leucine, and isoleucine give these cheeses a sweaty putrid aroma. Bloomy and washed rind cheeses were found to contain the halotolerant γ-proteobacteria, *Pseudoalteromonas*, a bacterial genus originally associated with marine environments. *Pseudoalteromonas* were found to possess a gene predicted to encode methionine-gamma-lyase (MGL), an enzyme that converts L-methionine to the aforementioned methanethiol. Among cheese microbes, this enzyme had only been found in *Brevibacterium linens* previously. *Pseudoalteromonas* spp. produce cold-adapted enzymes that participate in lipolysis and proteolysis, a feature that is regarded as beneficial in cheese aged and stored at low temperatures as it leads to the development of flavor compounds (Wolfe et al., 2014). Finally, with regard to flavor, pathways involved in the production of flavor and aroma compounds were captured through metagenomic analysis of Cotija cheese (Escobar-Zepeda et al., 2016). The microbes present encode enzymes with the potential to catabolise phenylalanine and branched chain amino acids to yield flavor compounds. The study also detected genes encoding enzymes involved in free fatty acids catabolism, such as carbonyl reductase I and phenol-2 monoxygenase (an enzyme responsible for the production alcohol from xylene) which results in products that impact a characteristic aroma to traditional cheeses. The genetic determinants for antimicrobial compounds such as bacteriocins produced by strains of LAB, which can play an important role in food biopreservation, were also present (Escobar-Zepeda et al., 2016).

Finally, shotgun metagenomic analysis of cheeses has also been employed to determine the microbial basis for cheese colors. Such analyzes of cheeses with a pink discoloration defect revealed the presence of genes that codes metabolic compounds responsible for the production of carotenoids, that were absent from control cheeses. The presence of this pathway was associated with microbes from the genus *Thermus*, which are renowned for their ability to produce carotenoids but had not been thought to be important in the industrial context of the cheese microbiota previously (Quigley et al., 2016). The aforementioned Bertuzzi et al. study also revealed that surface ripened cheese made from commercially available smear cultures have a greater potential for the production of carotenoids responsible for red/orange colors on the surface of cheeses, highlighting the key role of these smear culture-associated microbes in color development (Bertuzzi et al., 2018).

The next logical step beyond shotgun metagenomics (to assess functional potential) is the use of metatranscriptomics to identify genes that are actually expressed. Metatranscriptomic analysis of Camembert cheese, in which *G. candidum* and *Penicillium camemberti* play key roles in ripening, and the *de novo* assembly of the reads was employed to determine at what stage of cheese ripening are pathways associated with the production of flavor, aroma, and texture development expressed. The study found that these fungal species, already known to contribute to the appearance of the cheese, also play a key role in the development of sensory characteristics of Camembert (Lessard et al., 2014). Metatranscriptomics has also been employed to assess the impact of environmental conditions on gene expression in Caciocavallo Silano, a traditional Italian cheese. These investigations arose from observations that increasing the temperature and decreasing the humidity of the ripening room enhanced the growth of NSLAB in the cheese. Cheese ripened at a higher temperature for 20 days had a similar metabolic profile to that of cheese ripened at standard conditions over a long period, highlighting that increase in ripening temperature results in the rapid maturation of a cheese of quality, while helping the manufacturer to reduce operating costs and to increase the turnover of ripening rooms. The explanation for this reduced ripening time became apparent when it was established that higher temperatures boosted the expression of genes involved in proteolysis, lipolysis, amino acid and fatty acid catabolism and, thus, promoted the VOC related to flavor and aroma (De Filippis et al., 2016). Another such study focussed on a Reblochon-style French cheese, rind activity in which is mostly influenced by *S. thermophilus, L*. *delbrueckii* spp.*bulgaricus, G. candidum, D. hansenii*, and *Brevibacterium aurantiacum*. Metatranscriptomic analysis of this cheese during ripening showed the up-regulation of *S. thermophilus* genes associated with protein folding, sorting, degradation, and signal transduction. Transcription, translation as well as nucleotide and lipid metabolism was high among *L. delbrueckii*. Among the yeasts (*D. hansenii, G. candidum*) for *G. candidum* a large increase in the transcription of genes associated with carbohydrate, lipid, and amino acid metabolism was evident, for *D. hansenii* an increase in genes associated with the metabolism of other amino acids were noted. A decrease in the expression of gene associated with nucleotide metabolism was observed for both yeasts (Monnet et al., 2016). Metatranscriptomics can also be combined with other technologies to provide an even greater insight into the microbiology of cheese. Indeed, a combination of biochemical, metagenomic, and metatranscriptomic analysis has been used to study the microbiology of surface ripened cheese. In this particular study it became clear that dominant bacteria such as *L. lactis, Kluyveromyces lactis, D. hansenii, G. candidum, Corynebacterium casei*, and *Hafnia alvei* showed high levels of expression of transcripts related to primary (lactose metabolism, lipolysis, proteolysis) and secondary (catabolism of free amino acids and fatty acids) biochemical reactions (Dugat-Bony et al., 2015). It is anticipated that metatranscriptomic-based analysis of the cheese ripening process will become increasingly common in the near future.

## HTS reveals the presence of pathogenic and spoilage bacteria in cheese

HTS can also be employed to detect pathogenic or spoilage microbes in cheeses, milk, or in the production and processing environments. Numerous sources of contamination exist. These include cow feces and teat surfaces (Doyle et al., 2017a; Frétin et al., 2018) as well as bulk tanks and milking machines can also act as sources, such as in the case of the presence of *Enterococcus faecalis* in raw Herve cheese (Delcenserie et al., 2014).

The consequences of the presence of different microbes are various. The appearance of pink discoloration in cheese manifests through the appearance of pink patches at various locations within the ripened cheese block. As noted above, HTS revealed the presence of spoilage bacteria *Thermus thermophilus*, a species associated with the production of carotenoids, within defective cheeses (Quigley et al., 2016). The genera *Brevibacterium, Corynebacterium*, and *Microbacterium* have been associated with a red-brown defect in smear-ripened cheese, such as Fontina. Deacidification by Debaryomyces facilitates the growth of these bacteria on wooden shelves used for ripening (Guzzon et al., 2017). Analysis of Grana Padano cheeses that exhibited a blowing defect resulted in the detection of seven species of clostridia, i.e., *C. sporogenes, C. butyricum, C. disporicum, C. perfringens, C. difficile, C. sordelii*, and *C. tyrobutyricum*. Among these *C. tyrobutyricum* and *C. butyricum* were more abundant in blowing defect samples (Bassi et al., 2015). In another study, the presence of genera such as *Klebsiella, Morganella, Erwinia*, and *Acinetobacter*, i.e., taxa commonly found in soil and water, in cheese and curd samples of Plaisentif cheese was taken to represent poor hygiene conditions and the contamination of boilers or tools used in the early stages of processing (Dalmasso et al., 2016). Environmental contaminants, including *Escherichia* sp., *Enterobacter cowanii* and other *Enterobacteriaceae, Agrobacterium* sp., *Alicyclobacillus* sp., and *Propionibacterium acnes* were also detected in NWC used to make Italian cheeses (De Filippis et al., 2014) and the detection of *Pseudomonas* was observed in the intermediates of Mozzarella cheese production was suggested to be related to handling and hygiene conditions (Ercolini et al., 2012).

Another mechanism via which microbes can influence cheese quality is through the production of biogenic amines. Histamine, tyramine, putrescine, and cadaverine are the most common biogenic amines that occur in food, with their accumulation causing symptoms such as hypertension, headaches, palpitations, and vomiting in sensitive individuals. The presence of species such as *Lactobacillus curvatus, Enterococcus faecium*, and *Enterococcus faecalis* can result in the production of tyramine and *Lactobacillus buchneri* has been associated with the production of histamine. A HTS-based approach that specifically detects the decarboxylase genes that are responsible for biogenic amine production has been developed (O'Sullivan et al., 2015b).

One obvious concern relating to the use of DNA-based approaches to microbial detection is the possibility of false positives arising from the detection of DNA from dead microorganisms. The inclusion of steps involving the use of ethidium monoazide (EMA) IUPAC name: 3-amino-8-azido-5-ethyl-6-phenylphenanthridium bromide or propidium monoazide (PMA) IUPAC name: 3-Amino-8-azido-5-{3-[diethyl(methyl)ammonio]propyl}-6- phenylphenanthridinium to inactivate any DNA which is not sourced from living microbes provides a means of addressing this issue in the context of amplicon based sequencing at least (Rudi et al., 2005; Josefsen et al., 2010; Quigley et al., 2013; Porcellato and Skeie, 2016). Ultimately, while, currently, these investigations have been carried out to identify problematic microorganisms or the source thereof in academic laboratories, it is envisaged that the technology will evolve in a manner that will allow its use in quality assurance laboratories across the food industry.

## Conclusions

HTS is a powerful technique that can be used to provide a detailed insight into the microbiology of dairy related samples, including raw and pasteurized milk, cheese curd, whey, starter cultures, and cheese. The technology also allows a determination of the impact of seasonal variations, feed given to animals, source of milk, and other environmental factors relating to milk production and processing on the milk and cheese microbiota. Once the microbial community in cheese has been formed, HTS can then be employed to determine the role of the microbial population in factors such as the development of organoleptic properties. While there are very many advantages associated with the use of HTS, some barriers, such as the detection of DNA sequences from non-viable bacteria, cost effectiveness, and experience in bioinformatic analysis need to be overcome before these benefits can be applied extensively across industry. Furthermore, there is a need for the construction of a comprehensive dairy microbial gene catalogs to improve the analysis of HTS reads in the dairy environment (Almeida et al., 2014). However, given that solutions to these issues exist or are in development, it is hoped that any delays to the industrial application of this technology will be short.

## Author contributions

BY and PC prepared the manuscript and all co-authors contributed to editing and critical reviewing thereof.

### Conflict of interest statement

The authors declare that the research was conducted in the absence of any commercial or financial relationships that could be construed as a potential conflict of interest.

## Abbreviations

HTS: High Throughput Sequencing
NGS: Next Generation Sequencing
PDO: Protected designation of Origin
CIP: Cleaning-in-Place
NMC: Natural Milk Starter Cultures
NWC: Natural Whey Cultures
VOC: Volatile Components.
